# Neutrophilic dermatosis of the dorsal hands in a Mexican woman^☆^

**DOI:** 10.1016/j.abd.2022.12.015

**Published:** 2024-06-08

**Authors:** Carlos Barrera-Ochoa, Luis Enrique Cano-Aguilar, Hector Cantú-Maltos, Hector Proy-Trujillo, Nixma Eljure-López, María Elisa Vega-Memije

**Affiliations:** a Department of Dermatology, General Hospital “Dr Manuel Gea González”, Mexico City, Mexico; b Department of Dermatopathology, General Hospital “Dr Manuel Gea González”, Mexico City, Mexico; c Department of Dermatologic Surgery, Dermatology Center of Yucatán, Mérida, Mexico; d Department of Dermatology, Dermatology Center of Yucatán, Mérida, Mexico

Dear Editor,

A 46-year-old woman presented to our outpatient dermatology clinic with a one-month history of two spontaneous symmetric painful large plaques on the dorsal and palmar surfaces of her hands. On examination, we found two 5–8 cm wide plaques with an erythematous central surface, surrounded by a pale bullous halo and a purplish erythematous rim (Fig. 1). Past medical history was positive for chronic kidney disease treated with sodium bicarbonate and hydrochlorothiazide awaiting hemodialysis, and type 2 diabetes mellitus treated with pioglitazone. No previous trauma was informed, and no other treatments were previously applied. A biopsy of the lesion revealed an epidermal ulcer with leukocytoclastic vasculitis as well as a rich neutrophilic and lymphocyte infiltrate (Fig. 2).

**Fig. 1:**
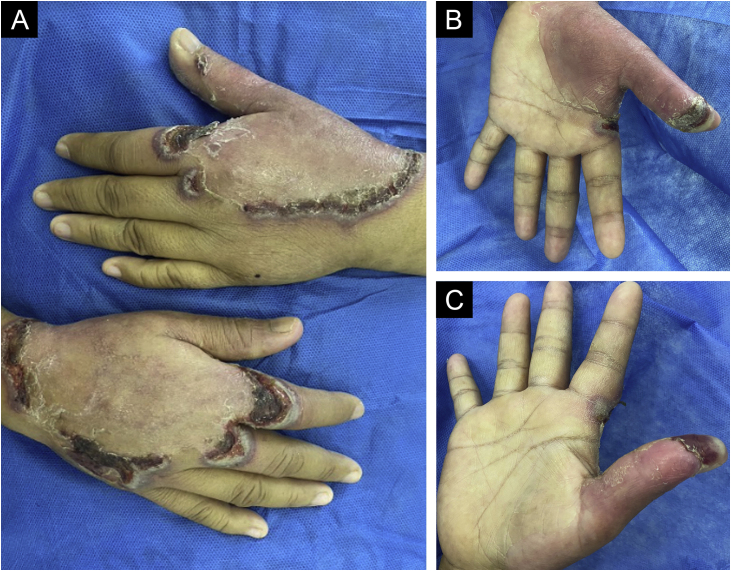
(A) Bilateral symmetric plaques on the dorsal surface of the hands with peripheral bullous halo, crusts, and a purplish erythematous rim. (B‒C) Palmar erythema with purplish bullous lesions on the thumbs.

**Fig. 2:**
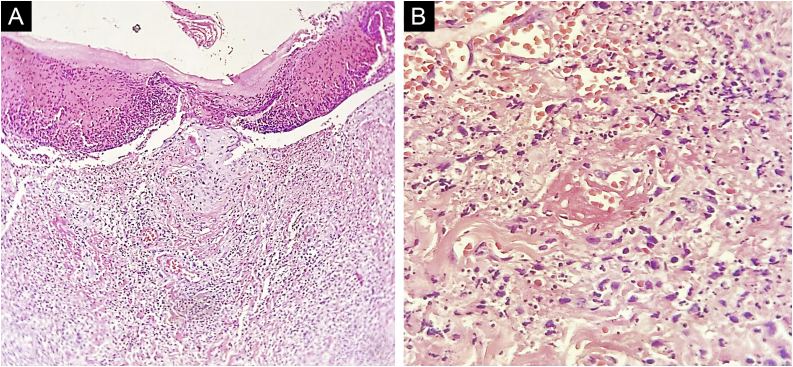
(A) Ulceration with entire epidermal necrosis (Hematoxylin & eosin, ×100). (B) Close-up view of the intense inflammatory infiltrate of neutrophils and lymphocytes with vasculitis through the dermis (Hematoxylin & eosin, ×400).

Neutrophilic Dermatosis of the Dorsal Hands (NDDH) is a rare variant of a neutrophilic dermatosis such as pyoderma gangrenosum and Sweet Syndrome.^1^ Few cases have been reported in the literature so far, and cutaneous involvement of the palms is seldomly described despite the radial dorsal surface of the hands as the most affected location, especially in the area between the thumb and index finger.^2^, ^3^ NDDH is characterized by erythematous plaques, bullae, pustules, and nodules that might present necrosis and ulceration, with progressive growth in a small period.^4^

A recent review of 123 cases of NDDH, showed a female predominance in 58% with a unilateral distribution in 78% and a mean age of 62 years. Due to its inflammatory pattern, serological studies may present leukocytosis (56.5%) and neutrophilia (83%) and mimic a soft skin tissue infection. Besides the latter, the differential diagnosis includes pyoderma gangrenosum, Sweet syndrome, and erythema elevatum diutinum.^3^, ^4^

The histological presence of an intense neutrophil infiltrate through the dermis with associated edema is considered an indicator of neutrophilic dermatosis. Other findings such as leukocytoclastic vasculitis may be found in the early stages of the disease due to endothelial damage but should not be considered as a criterion for making the diagnosis.

The pathogenesis of NDDH is not completely understood. Some authors have suggested trauma as a potential trigger and as with other neutrophilic dermatoses, multiple systemic associations have been described. Specifically, in NDDH, correlation with hematological or solid malignancies, rheumatological conditions, inflammatory bowel disease, and co-morbidities such as diabetes and chronic renal disease as in our patient, among others, should be evaluated.^5^

Although self-resolution might occur, treatment options for NNDH include systemic and topical corticosteroids, oral dapsone, and colchicine.^4^, ^5^ Our patient was treated with occlusive clobetasol for two weeks with a complete response. The patient refused to continue an extensive work-up and subsequent follow-up.

## Financial support

None declared.

## Authors’ contributions

Carlos Barrera-Ochoa: The study concept and design; data collection, or analysis and interpretation of data; writing of the manuscript or critical review of important intellectual content; data collection, analysis, and interpretation; effective participation in the research guidance; critical review of the literature; final approval of the final version of the manuscript.

Luis Enrique Cano-Aguilar: Writing of the manuscript or critical review of important intellectual content; data collection, analysis, and interpretation; effective participation in the research guidance; critical review of the literature.

Hector Cantú-Maltos: Writing of the manuscript or critical review of important intellectual content; data collection, analysis, and interpretation.

Héctor Proy-Trujillo: Effective participation in the research guidance; critical review of the literature; final approval of the final version of the manuscript.

Nixma Eljure-López: Effective participation in the research guidance; critical review of the literature; final approval of the final version of the manuscript.

María Elisa Vega-Memije: Critical review of the literature; final approval of the final version of the manuscript.

## Conflicts of interest

None declared.
